# B-cell-targeting therapies in podocytopathies

**DOI:** 10.1007/s00467-026-07297-4

**Published:** 2026-04-18

**Authors:** Carolina Bigatti, Paolo Cravedi, Andrea Angeletti

**Affiliations:** 1 Department of Medicine, Translational Transplant Research Center, Icahn School of Medicine at Mount Sinai, New York, NY USA; 2 Nephrology, Dialysis and Transplantation Unit, IRCCS Istituto Giannina Gaslini, Genoa, Italy

**Keywords:** Nephrotic syndrome, Minimal change disease, Focal segmental glomerulosclerosis, Rituximab, Daratumumab, Monoclonal antibodies, Recurrence

## Abstract

Podocytopathies, including minimal change disease and focal segmental glomerulosclerosis, represent the leading causes of nephrotic syndrome in children and adolescents. Although traditionally considered T-cell–mediated disorders, growing evidence over the last decade has reshaped this paradigm, highlighting a central role for B-cells, plasma cells, and humoral immune mechanisms in podocyte injury. The identification of pathogenic autoantibodies, particularly anti-nephrin IgG, and the involvement of complement activation have provided a strong mechanistic rationale for B-cell–targeted therapies. Rituximab, an anti-CD20 monoclonal antibody, has become an established steroid-sparing agent in steroid-dependent and frequently relapsing nephrotic syndrome, significantly reducing relapse rates and cumulative steroid exposure in pediatric patients. However, its efficacy is limited in steroid-resistant disease and in settings characterized by persistent autoantibody production or post-transplant recurrence. These limitations have prompted the exploration of next-generation anti-CD20 agents, such as obinutuzumab, which achieve deeper and more sustained B-cell depletion, as well as therapies targeting long-lived plasma cells, including anti-CD38 monoclonal antibodies. Emerging clinical data suggest that combined B-cell and plasma cell targeting may induce durable remission in refractory podocytopathies and recurrent post-transplant focal segmental glomerulosclerosis, while maintaining an acceptable safety profile. In parallel, novel strategies targeting the BAFF/APRIL axis and cellular therapies such as CAR-T cells are under investigation. This review summarizes current evidence on B-cell–directed therapies in podocytopathies, mostly in pediatrics, discusses unmet clinical needs, and outlines future perspectives toward precision, mechanism-based immunotherapy.

**Graphical abstract:**

A higher resolution version of the Graphical abstract is available as Supplementary information

**Figure:**
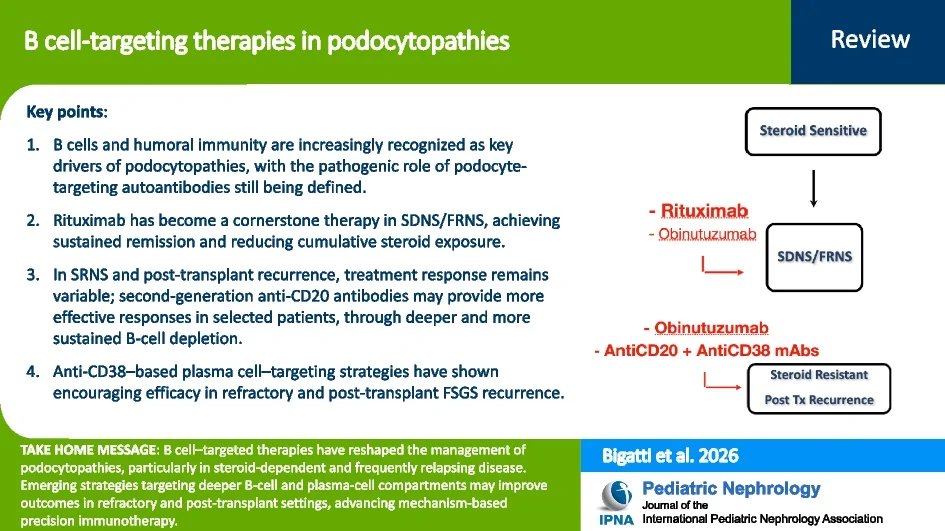

**Supplementary Information:**

The online version contains supplementary material available at https://doi.org/10.1007/s00467-026-07297-4.

## Introduction

Podocytopathies, including minimal change disease (MCD), focal segmental glomerulosclerosis (FSGS), and C1q nephropathy, represent the most common causes of nephrotic syndrome (NS) in children and adolescents. Despite increasing understanding of the underlying pathophysiological mechanisms, the current classification still relies primarily on the patient’s response to corticosteroid therapy, which remains the cornerstone of initial treatment [1].

While the majority of cases initially present as steroid-sensitive nephrotic syndrome (SSNS), a significant proportion subsequently evolve into steroid-dependent (SDNS) or frequently relapsing (FRNS) forms [2]. Conversely, approximately 10–20% of patients are classified as having steroid-resistant nephrotic syndrome (SRNS), a condition associated with a markedly increased risk of progression to chronic kidney disease and a high likelihood of post-transplant disease recurrence.

The classical paradigm of podocyte injury centered on T-cell–mediated mechanisms [3]. However, this concept has been challenged by initial serendipitous clinical observation that B-cell–depleting therapies, particularly anti-CD20 monoclonal antibodies such as rituximab, can induce sustained remission in patients with MCD or FSGS [4]. This prompted a re-evaluation of the role of B-cells and humoral immunity in disease pathogenesis. Evidence generated over the last decade supports a central involvement of B-cells, plasma cells, and circulating autoantibodies in mediating podocyte injury [5, 6]. In particular, antinephrin autoantibodies have been detected in 70–90% of patients during active nephrotic syndrome. These antibodies directly target nephrin at the slit diaphragm, inducing its abnormal phosphorylation [7] leading to a signaling cascade resulting in actin cytoskeletal remodeling, nephrin endocytosis [8], and ultimately podocyte foot process effacement and proteinuria. More recently, IgG targeting other components of the slit diaphragm has been proposed to play a pathogenic role in MCD and in a subset of FSGS patients [9]. Recent single-cell transcriptomic analyses in children with active idiopathic nephrotic syndrome have further demonstrated an expansion of extrafollicular B-cell populations, including atypical B-cells and short-lived antibody-secreting cells, supporting an extrafollicular origin of humoral immune activation in disease flares [10].

Emerging evidence indicates that activation of the classical complement pathway may contribute to glomerular injury in FSGS. Naturally occurring IgM antibodies, capable of recognizing glomerular endothelial or mesangial epitopes [11, 12], have been implicated in initiating complement activation and subsequent deposition within the renal parenchyma. Elevated levels of complement activation products in both serum and urine during active disease phases further support the involvement of complement-mediated mechanisms, via both the classical and alternative pathways, in the pathogenesis of these disorders [13, 14].

Collectively, these findings shift the paradigm from a solely T-cell–driven process to a complex immunological network involving B-cell–derived autoantibodies and complement-mediated damage. The identification of these humoral components has opened new therapeutic avenues, particularly the targeting of B-cells (e.g., CD20^+^ cells with rituximab or obinutuzumab) and plasma cells (e.g., anti-CD38 therapies like daratumumab). This mechanistic insight is also driving the development of disease-specific biomarkers, with antinephrin IgG now being explored for diagnosis, relapse prediction, and response monitoring.

Herein, we review B-cell–targeted therapies in podocytopathies, discussing current evidence, emerging strategies, and unmet clinical needs in this evolving therapeutic landscape.

## Definition of podocytopathies and clinical implications

Although in pediatric nephrology the term *nephrotic syndrome* is commonly used as a unifying clinical diagnosis, it encompasses multiple histological and pathophysiological entities. Renal biopsy is not routinely performed in children, particularly in steroid-sensitive forms, and the underlying lesion is therefore often presumed to be MCD [15]. In contrast, adults presenting with a similar clinical phenotype are more likely to undergo kidney biopsy, reflecting a broader differential diagnosis that includes not only MCD but also FSGS, membranous nephropathy (MN), and less common variants such as C1q or IgM nephropathy. These latter entities are characterized by dominant mesangial deposition of C1q or IgM, respectively, and may occur in children and young adults as well, often overlapping with MCD or FSGS patterns.

MCD is generally associated with excellent response to steroids, in contrast, FSGS typically has a less favorable prognosis due to the high risk of steroid resistance and progression to chronic kidney disease, reflecting more severe and irreversible podocyte loss.

To bridge the differences in terminology and emphasize the shared pathogenic substrate of podocyte injury, many experts now adopt the term primary podocytopathies for identifying glomerular diseases caused by initial podocyte injury or dysfunction that lead to NS, which encompasses both MCD and FSGS regardless of age or clinical presentation [16]. This unified terminology is supported by recent guidelines, which base treatment decisions not on histology, but rather on the response to steroids, categorizing patients as SS or SR in both pediatric and adult settings.

Outcome data further support this approach. While children generally exhibit better renal survival than adults, this advantage is largely due to the higher prevalence of MCD in pediatric cohorts. When analyses are adjusted for histologic subtype, the impact of age on long-term kidney outcomes is significantly attenuated. In other words, histology, not age, should be considered as the main prognostic factor. However, it is important to note that a diagnosis of MCD does not automatically imply a benign course. Some patients with histologically confirmed MCD experience frequent relapses, develop steroid dependence or resistance, and may even progress to chronic kidney disease. Across all age groups, patients with FSGS consistently show worse renal outcomes than those with MCD. Therefore, the term podocytopathies not only reflects the underlying biology but also facilitates a unified clinical framework across age groups, centered on both response to treatment and histologic risk.

## Rituximab

Rituximab is a chimeric monoclonal antibody targeting the CD20 antigen expressed on pre-B and mature B lymphocytes. It was originally approved for the treatment of hematologic malignancies and rheumatologic conditions, and its mechanism of action involves multiple cytotoxic pathways, including complement-dependent cytotoxicity (CDC), antibody-dependent cellular cytotoxicity (ADCC), and the induction of phagocytosis and apoptosis. Rituximab leads to effective depletion of both naïve and memory B-cells, while sparing long-lived plasma cells that do not express CD20.

Its potential role in nephrotic syndrome was first suggested in 2004, when a child with SDNS secondary to MCD achieved remission of proteinuria following rituximab administration for idiopathic thrombocytopenic purpura [16, 17]. This incidental observation was followed by additional reports describing the resolution of post-transplant FSGS recurrence in patients treated with rituximab for post-transplant lymphoproliferative disorders (PTLD), suggesting a possible therapeutic effect on podocyte diseases [18].

These early anecdotal findings paved the way for more systematic investigations. Over the past two decades, several randomized controlled trials (RCTs) and prospective cohort studies have confirmed the efficacy of rituximab in maintaining remission and reducing relapse rates in children with SDNS and FRNS. However, treatment response differs substantially between SDNS/FRNS and SRNS, warranting a distinct evaluation of available evidence.

### Rituximab in SDNS/FRNS

The multicenter, double-blind RCT by Iijima et al*.* demonstrated that a single course of rituximab (4 weekly doses of 375 mg/m^2^) significantly prolonged the median relapse-free period compared to placebo (267 vs. 101 days; HR 0.27, 95% CI 0.14–0.53) in children with complicated SDNS/FRNS [19]. The NEMO trial (Ruggenenti et al.) [20] evaluated the efficacy of rituximab followed by withdrawal of oral immunosuppressive agents in both pediatric and adult patients with steroid-dependent or frequently relapsing nephrotic syndrome (SDNS/FRNS). Rituximab significantly reduced relapse rates and overall steroid exposure. Moreover, in the pediatric cohort, treatment was associated with stabilization of growth trajectories and a significant improvement in growth velocity (Table 1). 

Repeated rituximab courses have shown increasing efficacy over time. In a large international multicenter study of 346 children with FRNS or SDNS, the median relapse-free period improved from 10 months after the first course to 12–16 months with subsequent courses, with a progressive reduction in relapse risk [21].

More recently, a retrospective multicenter study on 183 adults with a histological diagnosis of primary MCD or FSGS treated with rituximab reported 45% and 36% of remission at 1 and 3 years of follow-up, respectively. The cohort was largely composed of steroid-sensitive forms (68%), supporting that rituximab may be considered an effective option in this specific setting also in adults [22] (Table 1).

**Table 1:** Main clinical studies describing the use of rituximab in primary podocytopathies

Reference	*N*	F/U (mo)	Study design	Dose	CR (%)
SDNS/FRNS					
Ravani et al*.* [53]	46	12	Off–on trial	375 mg/m^2^ (1–5 doses)	48
Iijima et al*.* [18]	48	12	RCT	375 mg/m^2^ (4 doses) vs. placebo	81
Ruggenenti et al*. *[19]	30	12	Off–on trial	375 mg/m^2^ (1–2 doses)	100
Ravani et al*.* [54]	30	24	RCT	375 mg/m^2^ vs. steroid	66 at 12mo, 34 at 24mo
Chan et al*.* [20]	346	24	Multicenter, retrospective cohort study	375 mg/m^2^ or 750 mg/m^2^. Multiple courses (2 to more than 7)	-
Gauckler et al. [21]	183	36	Retrospective	1000 mg (1 or 2 doses) or 375 mg/m^2^ (1 to 4 doses)	Initial CR/PR at 6 mo: 82%; long-term relapse-free survival among responders: 55% at 3 years
Laslandes et al*.* [22]	134	36	Multicenter retrospective cohort	Variable regimens; maintenance RTX at 6–12 mo	Relapse rate reduced from 1.02 to 0.20 per pt-year; maintenance RTX associated with lower relapse risk (HR 0.35)
SRNS					
Magnasco et al. [25]	31	12	RCT	375 mg/m^2^ (2 doses) vs. prednisone and calcineurin inhibitors	6 delayed-resistant patients achieved remission (3 RTX, 3 control)
Chan et al*.* [26]	246	32.4 (mean)	Retrospective	From 375 to 1500 mg/m^2^	-
Gauckler et al*.* [21]	183	36	Retrospective	1000 mg (1 or 2 doses) or 375 mg/m^2^ (1 to 4 doses)	-

*CR* complete response, *FRNS* frequently relapsing nephrotic syndrome, *F/U* follow-up, *HR* hazard ratio, *mo* months, *PR* partial remission, *RCT* randomized controlled trial, *RTX* rituximab, *SDNS* steroid-dependent nephrotic syndrome

In adults with idiopathic nephrotic syndrome, growing evidence supports the role of rituximab not only for relapse treatment but also as a maintenance strategy. In a large French multicenter cohort, maintenance rituximab administered between 6 and 12 months after induction was associated with a significantly lower risk of relapse, without an increased rate of severe infections [4].

Overall, 12-month remission rates in SDNS/FRNS range from approximately 60 to 80% across different pediatric cohorts, confirming rituximab as an effective steroid-sparing strategy in this population. 

### Rituximab in SRNS

On the other hand, the efficacy of rituximab in SRNS compared to common protocols, such as calcineurin inhibitors (CNI) and plasma exchange, is less clear. Bagga et al. treated five children with SRNS using rituximab, which promoted complete and partial remission in three and two patients, respectively [23]. A subsequent case series reported stable remission in approximately half of 33 pediatric patients with steroid-resistant nephrotic syndrome treated with rituximab after 12 months of follow-up [24]. In 2012, Magnasco et al. conducted an RCT in 31 children with nephrotic syndrome resistant to combined calcineurin inhibitor and steroid therapy. Patients were assigned to receive either two monthly infusions of rituximab (375 mg/m^2^) in addition to ongoing therapy or continued treatment with CNI and steroids alone. The addition of rituximab did not result in a higher rate of remission [25]. More recently, in a multicentric retrospective analysis on 246 pediatric patients with SRNS (57% FSGS), the remission rates (CR/PR) were 39% at 2 years of follow-up [26] (Table 1).

Overall, observational studies suggested a complete remission rate of about 20–30% of cases treated with rituximab, but the limited controlled randomized studies did not support the superiority of rituximab compared to other treatments.

### Safety

Rituximab is generally well tolerated in pediatric podocytopathies. The most frequent adverse events are infusion-related reactions, occurring in approximately 5–10% of cases. These reactions, such as fever, rash, and bronchospasm, are typically mild and transient and can be minimized through slow infusion protocols and premedication with corticosteroids, antihistamines, paracetamol, and bronchodilators [27].

Serious adverse events are rare and limited to isolated case reports, including pulmonary fibrosis and myocarditis. Progressive multifocal leukoencephalopathy has been described but is not clearly attributable to rituximab, as it has also been reported with other immunosuppressive agents.

The chimeric nature of rituximab can elicit antidrug antibodies (ADA), potentially reducing efficacy or increasing the risk of infusion reactions. Interpretation of ADA kinetics remains complex, as antibody titers may decline over time and results are influenced by the timing of sampling in relation to rituximab exposure. Previous evidence suggests that high baseline ADA titers prior to subsequent rituximab administration may be associated with shorter B-cell depletion and earlier relapse, as well as increased risk of infusion-related adverse events [28]. However, data remain limited and heterogeneous. In our previous study [29], baseline ADA levels prior to rituximab re-exposure were negative, precluding a formal evaluation of their impact on clinical response.

Overall, while ADA formation has been reported particularly after repeated exposure, its true clinical relevance in pediatric podocytopathies remains to be fully elucidated and warrants further prospective investigation.

A relevant concern is the risk of hypogammaglobulinemia, particularly after repeated rituximab administrations. In a large pediatric cohort treated for SDNS, approximately 35% of patients developed hypogammaglobulinemia, which often persisted for several months [30]. Reported cases are usually mild to moderate (IgG 400–600 mg/dL), whereas severe hypogammaglobulinemia (< 400 mg/dL) appears less frequent (10–15%), but may require closer monitoring [31–33]. Identified risk factors include younger age at treatment, low baseline IgG levels, repeated rituximab courses, and concomitant or prolonged exposure to other immunosuppressive agents [33]. While asymptomatic in most cases, this condition may predispose to infections, particularly of the respiratory tract. However, the clinical significance of persistent low IgG levels remains uncertain, as many patients remain infection-free. In most series, IgG levels gradually recover within 6–12 months following B-cell reconstitution, although prolonged hypogammaglobulinemia has been described in a minority of patients [34]. Regular monitoring of serum immunoglobulin levels is recommended, and immunoglobulin replacement therapy should be considered in selected cases with recurrent or severe infections.

Late-onset neutropenia, including rare cases of agranulocytosis, has also been reported after rituximab therapy. Although uncommon and typically transient, late-onset neutropenia usually occurs several weeks to months after infusion and may increase the risk of bacterial infections [35, 36]. Therefore, periodic blood count monitoring is advisable, particularly in patients receiving repeated courses or concomitant immunosuppressive therapy.

Of note, rituximab may increase the risk of Hepatitis B Virus (HBV) reactivation, even in patients with previously resolved infections, potentially causing severe liver failure or death, necessitating universal HBV screening before treatment and prophylactic antiviral therapy for high-risk individuals.

Taken together, these findings support the use of rituximab as an effective steroid-sparing agent in patients with steroid-dependent and frequently relapsing podocytopathies. Its ability to induce and maintain remission, reduce cumulative steroid exposure, and limit the need for additional immunosuppressive agents make it an attractive option in this population. Notably, clinical experience suggests that response to rituximab may be particularly favorable in older children and adolescents, typically above 10 years of age, although this observation requires further validation in age-stratified trials.

### The issue of rituximab dosing

The optimal dosing regimen of rituximab remains a matter of ongoing debate. Current KDIGO guidelines recommend a single dose of 375 mg/m^2^; however, available evidence remains insufficient to support a definitive recommendation regarding the number of doses per treatment course [37].

Rituximab was initially administered in nephrological settings using the same four-weekly 375 mg/m^2^ dosing schedule developed for B-cell malignancies. Over time, alternative regimens have been adopted. These include two-dose regimens (e.g., 750 mg/m^2^ × 2), commonly used in systemic autoimmune diseases, and single-dose protocols. However, a lack of randomized comparisons between dosing strategies has historically limited consensus, resulting in significant variability across centers worldwide. While earlier data suggested higher cumulative doses (1500 mg/m^2^) [19] might prolong remission, more recent evidence indicated that a single infusion of 375 mg/m^2^ can be equally effective as multiple infusions *per* course [30]. Very low-dose regimens (e.g., 100 mg/m^2^) may be insufficient to ensure sustained remission, as indicated by higher relapse rates [38, 39].

A large international retrospective study involving 511 children with SDNS/FRNS compared six real-world treatment regimens, stratified by rituximab dose, low (375 mg/m^2^), medium (750 mg/m^2^), and high (1125–1500 mg/m^2^), and by the presence or absence of concomitant maintenance immunosuppression. The poorest outcomes were observed in patients who received low-dose rituximab without maintenance therapy. In contrast, all other regimens achieved comparable relapse-free survival, ranging from 10.9 to 14.0 months. These findings support the use of moderate total rituximab doses (375–750 mg/m^2^) in combination with maintenance immunosuppression, rather than relying on dose escalation of rituximab alone [40].

## Other anti-CD20 monoclonal antibodies

### Ofatumumab

Ofatumumab is a fully humanized monoclonal antibody targeting the CD20 antigen. Compared to rituximab, ofatumumab binds to a distinct, more membrane-proximal epitope on CD20 and demonstrates higher affinity and enhanced CDC in vitro. These properties initially raised interest in its potential clinical superiority, particularly in patients unresponsive to rituximab.

In the nephrological field, ofatumumab was first tested in five children with SRNS refractory to rituximab. The treatment regimen consisted of an initial dose of 300 mg/1.73 m^2^, followed by five weekly infusions of 2 g/1.73 m^2^. At 12 months of follow-up, complete and partial remission were achieved in three and two patients, respectively [41]. These promising results, however, were not consistently replicated in subsequent studies.

In a report of two cases of steroid- and rituximab-resistant nephrotic syndrome, a single dose of ofatumumab was able to achieve remission. Wang et al. treated five patients with a similar protocol; one patient discontinued due to severe infusion reactions, while the remaining four achieved either complete (*n* = 3) or partial (*n* = 1) remission [42]. In contrast, a more recent RCT of ofatumumab (1.5 g/1.73 m^2^) versus placebo in multidrug-resistant nephrotic syndrome (MRNS) was interrupted after 13 of the planned 50 children for futility [43]. Ofatumumab has also been proposed in isolated cases of post-transplant nephrotic syndrome recurrence [44]. However, in the largest reported series involving six children resistant to prior treatments, no complete remissions were observed, with partial response in only half of the cases. Based on the limited available data, the safety profile of ofatumumab in podocytopathies appears comparable to that of rituximab, with infusion-related reactions and hypogammaglobulinemia representing the most commonly reported adverse events [45]. However, the relatively small number of treated patients and the lack of long-term follow-up substantially limit definitive conclusions regarding infection risk and sustained immunologic safety.

Given these mixed results and the absence of consistent superiority over rituximab, attention has recently shifted toward newer anti-CD20 agents with distinct mechanisms of action, such as obinutuzumab, which may offer enhanced B-cell depletion and improved clinical outcomes in refractory podocytopathies.

### Obinutuzumab

Obinutuzumab is a humanized, glycoengineered type II anti-CD20 monoclonal antibody, specifically designed to enhance immune-mediated cytotoxicity. Unlike rituximab, which binds CD20 in a type I conformation and primarily induces CDC, obinutuzumab binds in a type II orientation, favoring direct B-cell apoptosis and stronger ADCC through enhanced recruitment of effector cells, such as macrophages and natural killer cells. In addition, compared with rituximab, obinutuzumab achieves more effective B-cell depletion within secondary lymphoid tissues, where rituximab penetration and activity are relatively limited. These mechanistic differences translate into deeper and more sustained B-cell depletion, including naïve, memory B-cell, and resident B-cell subsets [46].

Clinical experience with obinutuzumab in patients with podocytopathies remains limited but promising. In small series of children and adults with steroid-resistant or rituximab-refractory nephrotic syndrome, obinutuzumab has led to persistent complete or partial remission, even in cases with early relapse following rituximab exposure [47, 48]. The superior efficacy may be linked not only to its distinct pharmacodynamic properties but also to its lower immunogenicity. Unlike rituximab, obinutuzumab appears less likely to induce antidrug antibodies, which can compromise the efficacy of subsequent infusions and accelerate B-cell reconstitution.

Beyond steroid-resistant cases, obinutuzumab has also shown activity in SS forms, including frequently relapsing and steroid-dependent MCD, as well as in MN. In a recent retrospective pediatric series, obinutuzumab resulted in longer relapse-free survival compared to rituximab in patients with SDNS or FRNS previously treated with rituximab [49]. These observations support its potential use not only as a rescue therapy but also as a first-line option in patients with a history of rituximab failure or suspected immunogenicity. Given that clinical trials in nephrotic syndrome are still ongoing, available safety data in podocytopathies are currently limited to small case series and real-world experiences. Based on these preliminary reports, obinutuzumab appears to have a safety profile comparable to other anti-CD20 agents, with infusion-related reactions and hypogammaglobulinemia being the most frequently described adverse events. Moreover, data from clinical trials in other immune-mediated diseases, including systemic lupus erythematosus, suggest an overall acceptable safety profile [50]. However, robust conclusions regarding long-term infection risk and sustained immunologic effects in podocytopathies will require confirmation from prospective trials and broader worldwide clinical experience.

Although further prospective trials are needed, especially with deeper immune phenotyping, current evidence suggests that obinutuzumab could represent a valuable therapeutic option for the management of difficult-to-treat podocytopathies.

Given that clinical trials in nephrotic syndrome are still ongoing, available safety data in podocytopathies are currently limited to small case series and retrospective analyses. However, the risk of long-term infection and sustained immunologic effects needs to be established in larger cohorts of patients with podocytopathies followed for long periods [51].

Despite the therapeutic benefits of anti-CD20 monoclonal antibodies, these agents selectively target mature B-cells, leaving long-lived plasma cells, which do not express CD20, largely unaffected. This limitation may contribute to persistent disease activity or relapses in certain patients, particularly those with sustained autoantibody production. Long-lived plasma cells residing in survival niches may continue to secrete pathogenic antibodies despite complete peripheral B-cell depletion, while autoreactive B-cell precursors may repopulate the immune system after treatment. These observations have spurred growing interest in therapies directed at plasma cell markers, which may allow deeper modulation of the humoral immune response in resistant forms of podocytopathy.

## Anti-CD38 monoclonal antibody

The limited efficacy of anti-CD20 therapies in complex or refractory podocytopathies has prompted growing interest in therapeutic strategies targeting long-lived plasma cells, which are resistant to CD20-directed agents and constitute a major source of autoreactive IgM. These plasma cells express high levels of CD38, a transmembrane glycoprotein involved in immune regulation, thus providing a rationale for targeting them with anti-CD38 monoclonal antibodies such as daratumumab.

A first proof-of-concept was provided by Dossier et al., who treated 14 children with SD/FR podocytopathies who had relapsed following prior rituximab therapy. Patients received a combination of obinutuzumab and daratumumab, which resulted in prolonged remission compared to rituximab alone [52]. However, the specific contribution of daratumumab over obinutuzumab alone remains uncertain, given the relatively mild disease course in this cohort.

More recently, a cohort study extended this therapeutic approach to patients with multidrug-resistant and multidrug-dependent podocytopathies, clinical phenotypes associated with increased risk of disease progression and cumulative treatment-related toxicity. In a cohort of 23 patients, a single infusion of rituximab and daratumumab induced remission of proteinuria in the majority of multidrug-resistant cases and enabled complete withdrawal of immunosuppressive therapy in all multidrug-dependent patients [53]. Clinical response correlated with depletion of CD19⁺ B-cell, CD38⁺ plasma cells, and circulating IgM levels [54]. Notably, the combined therapy was well tolerated, with no serious adverse events and only mild infusion-related reactions reported.

Together, these findings support the rationale for dual B-cell and plasma cell targeting in severe podocytopathies, providing a new therapeutic option where conventional immunosuppression fails. Prospective trials are warranted to define their role in future treatment algorithms.

## Post-transplant FSGS recurrence

FSGS frequently recurs after kidney transplantation, often within hours to days after transplant. Plasma exchange (PEX) and rituximab remain the most widely used therapies for post-transplant recurrence. However, their efficacy is inconsistent, and long-term use of PEX is burdensome and poorly tolerated. Moreover, a significant proportion of patients either do not respond or become dependent on repeated treatments [54–56].

In recent years, attention has shifted toward combination therapies targeting both B-cell and plasma cells, particularly in cases refractory to conventional approaches. On this basis, next-generation biologics such as obinutuzumab and daratumumab have been employed, either sequentially or in combination. Early reports suggest that these regimens can induce sustained remission and allow discontinuation of PEX in patients with rituximab-resistant disease, though data remain limited to small case series. Several case series have reported encouraging outcomes with these combination approaches in refractory post-transplant FSGS [57]. We recently reported a series of five patients with early post-transplant recurrence unresponsive to both PEX and rituximab who received combined therapy with rituximab and daratumumab, achieving complete remission in all cases [58]. The treatment was well tolerated, and flow cytometry analysis revealed a reduction in circulating plasma cells, supporting a mechanistic link between plasma cell activity and disease recurrence.

Similarly, case reports and small series have described the successful use of obinutuzumab plus daratumumab in patients with rituximab-resistant disease. In these patients, remission was typically achieved within weeks after treatment, which allowed PEX discontinuation and sustained proteinuria remission [59]. Although numbers remain small, these experiences suggest that dual targeting of CD20⁺ and CD38⁺ cells may provide a more effective strategy to suppress the production of pathogenic circulating factors in selected patients.

## Future perspectives

### BAFF/APRIL-targeted therapies

BAFF (B-cell activating factor) and APRIL (A Proliferation-Inducing Ligand) are members of the TNF ligand superfamily that play essential and complementary roles in B-cell homeostasis. BAFF primarily supports the maturation and survival of transitional and naïve B-cells, whereas APRIL contributes to the maintenance of long-lived plasma cells and sustained immunoglobulin production. Both ligands exert their effects through receptors expressed at distinct B-cell differentiation stages, including BAFF-R, TACI, and BCMA (B-cell maturation antigen). BCMA is predominantly expressed on plasma cells, where its engagement by BAFF or APRIL promotes survival and prolonged antibody secretion.

Aberrant activation of the BAFF–APRIL–BCMA axis has been implicated in several antibody-mediated autoimmune diseases, such as systemic lupus erythematosus (SLE) and rheumatoid arthritis, by enabling the persistence of autoreactive B-cell and plasma cells [60]. Therefore, targeting this pathway represents an attractive therapeutic approach for antibody-mediated autoimmune diseases, including glomerulonephritides.

Belimumab, a fully human monoclonal antibody that selectively neutralizes soluble BAFF, has demonstrated efficacy in SLE and lupus nephritis but has not produced consistent benefit across broader autoimmune kidney diseases. Its limited impact may reflect incomplete suppression of plasma cell survival, as BAFF inhibition alone does not affect APRIL-mediated signaling.

To achieve broader immunomodulation, recombinant fusion proteins containing the extracellular domain of TACI linked to an IgG Fc fragment have been developed to neutralize both BAFF and APRIL. These dual inhibitors reduce the survival of both B-cells and plasma cells and have shown promising results in autoimmune conditions such as SLE and IgA nephropathy [61]. Although clinical trials testing these agents in nephrotic syndrome or other podocytopathies are not yet available, the central role of autoreactive B-cells and circulating antibodies in podocyte injury provides a strong mechanistic rationale to evaluate BAFF–APRIL blockade in these diseases.

### Chimeric antigen receptor (CAR) T cells

Chimeric antigen receptor (CAR) T cells are under investigation as a novel therapeutic strategy in autoimmune diseases. Unlike monoclonal antibodies, CAR-T cells targeting B-cell antigens such as CD19 can induce deep and sustained B-cell depletion, potentially offering benefit in patients with refractory or relapsing nephrotic syndrome unresponsive to conventional therapies, including rituximab. Clinical experience in podocytopathies remains limited. A recent case report described the use of anti-CD19 CAR-T cells in a sensitized patient with recurrent FSGS, resulting in reduced anti-HLA antibody levels but failing to prevent disease recurrence, highlighting both the promise and current limitations of this approach [62]. Most clinical data on CAR-T therapy in autoimmunity come from SLE, where early-phase trials have shown sustained drug-free remission [63]. A clinical trial is currently ongoing to evaluate the safety and efficacy of anti-CD19 CAR-T cells in patients with multidrug-resistant or steroid-resistant NS (NCT06842589).

## Combined therapeutic approaches

As nephrologists, we may need to begin rethinking our therapeutic strategies for podocytopathies by drawing inspiration from hematology and oncology, where combination therapy has become a cornerstone of modern treatment paradigms. In these fields, the integration of agents targeting distinct yet complementary pathways has significantly improved disease control, allowing for more durable responses while carefully managing toxicity. Given that many immunomodulatory agents now entering nephrology were originally developed and validated in those disciplines, it is reasonable to ask whether similar combination-based approaches could enhance outcomes in glomerular diseases.

Rather than expecting novel agents to deliver transformative benefits as monotherapies, their integration with existing treatments, particularly anti-CD20 antibodies, which may serve as the therapeutic backbone, could offer a more effective and individualized strategy. In this context, the addition of agents targeting long-lived plasma cells or other components of the humoral immune response may further deepen and sustain immunologic remission, particularly in patients with complex or refractory disease. The recent clinical experience combining anti-CD20 and anti-CD38 therapy underscores how such regimens can increase the depth of response while maintaining an acceptable safety profile.

Beyond dual biologic strategies, combination regimens incorporating conventional immunosuppressive agents remain highly relevant in clinical practice. In SDNS/FRNS, rituximab is frequently administered alongside calcineurin inhibitors or mycophenolate mofetil, either as bridging therapy or maintenance treatment. As mentioned above, comparative data suggest that while different rituximab dosing schedules (ranging from single to multiple infusions) may achieve similar relapse prevention, the addition of mycophenolate mofetil to a single rituximab infusion may prolong remission compared with rituximab monotherapy [63]. These findings highlight the importance of optimizing combination protocols rather than focusing solely on the intensification of dosing of biologics.

Looking ahead, the adoption of rational, hypothesis-driven combination regimens could mark a critical step forward in the management of podocytopathies. Realizing this vision will require a cultural shift within the nephrology community, broader use of immunologic tools to guide therapy, and a commitment to structured, evidence-based frameworks that balance innovation with safety. If embraced thoughtfully, this evolution could move the field closer to true precision immunotherapy in nephrology.

## Conclusion

Podocytopathies are a biologically and clinically heterogeneous group of glomerular disorders in which B-cell–mediated mechanisms have emerged as central pathogenic drivers. Over the past two decades, B-cell–targeted therapies have significantly improved the management of SDNS/FRNS. Nonetheless, important challenges remain, including treatment resistance, relapse after B-cell reconstitution, and disease recurrence following kidney transplantation.

The growing understanding of humoral immune mechanisms in glomerular diseases has led to the development of novel therapeutic strategies that target B-cell and plasma cells at multiple levels. As these approaches continue to advance, future treatment paradigms are expected to move toward personalized, mechanism-based strategies incorporating immune profiling, biomarker-driven decisions, and rational combination regimens. Translating these immunological insights into clinical practice will be crucial to improving long-term outcomes in patients with difficult-to-treat podocytopathies.

## Supplementary Information

Below is the link to the electronic supplementary material.

ESM 1 Graphical abstract (PPTX 172 KB)
